# Genomic screening in family-based association testing

**DOI:** 10.1186/1471-2156-6-S1-S115

**Published:** 2005-12-30

**Authors:** Amy Murphy, Matthew B McQueen, Jessica Su, Peter Kraft, Ross Lazarus, Nan M Laird, Christoph Lange, Kristel Van Steen

**Affiliations:** 1 Department of Biostatistics, Harvard School of Public Health, Boston, MA 02115, USA; 2 Department of Epidemiology, Harvard School of Public Health, Boston, MA 02115, USA; 3 Channing Laboratory, Harvard Medical School, Boston, MA 02115, USA

## Abstract

Due to the recent gains in the availability of single-nucleotide polymorphism data, genome-wide association testing has become feasible. It is hoped that this additional data may confirm the presence of disease susceptibility loci, and identify new genetic determinants of disease. However, the problem of multiple comparisons threatens to diminish any potential gains from this newly available data. To circumvent the multiple comparisons issue, we utilize a recently developed screening technique using family-based association testing. This screening methodology allows for the identification of the most promising single-nucleotide polymorphisms for testing without biasing the nominal significance level of our test statistic. We compare the results of our screening technique across univariate and multivariate family-based association tests. From our analyses, we observe that the screening technique, applied to different settings, is fairly consistent in identifying optimal markers for testing. One of the identified markers, TSC0047225, was significantly associated with both the ttth1 (p = 0.004) and ttth1-ttth4 (p = 0.004) phenotype(s). We find that both univariate- and multivariate-based screening techniques are powerful tools for detecting an association.

## Background

Over the last few years, the field of statistical genetics has been the subject of challenging and exciting evolutions. As a result of human genetic sequencing, not only has data quality improved substantially, but the sheer volume of available data has vastly increased. The success of genome-wide association studies will depend upon whether the increase in numbers of single-nucleotide polymorphisms (SNPs) can be translated into an increase in the overall power to detect genotype × phenotype associations, or whether this potential increase in power will be diluted by the problem of multiple comparisons. In Van Steen et al. [1], the authors proposed that the multiple comparisons issue in genome × wide association testing could be addressed using the screening tools initially developed by Lange et al. [2,3]. This screening methodology finds the genotype × phenotype combinations with the most power to detect an association, without biasing any subsequently computed test statistic. In our paper, we apply this methodology, using the software package PBAT (pedigree-based association testing) [4] to sift through SNP data from the Collaborative Study on the Genetics of Alcoholism (COGA).

## Methods

### Dataset

In our analysis, the data was restricted to the genotypes from the Affymetrix SNP panel. This SNP data, along with quantitative trait information for approximately 1,613 subjects, comprised our dataset. The 1,613 subjects come from 143 families, with a mixture of large and small pedigrees. Using chromosome 4 as a template for the genome-wide screening techniques, we investigate the association between the 786 Affymetrix SNPs on chromosome 4, and the four measures corresponding to the "late" time window from the Visual Oddball Experiment, ttth1-ttth4. These four measurements correspond to electrode placements on the midline, central midline, and parietal midline channels, respectively. The phenotypes were transformed to standard normal scores for the analysis.

While there appears to be no documented research supporting an association between chromosome 4 (or another chromosomal regions) and the "late" time window measurements, several studies have proposed a region of linkage for the electroencephalogram (EEG) phenotype on chromosome 4 [5-7]. Furthermore, EEG demonstrates some correlation with the ttth phenotypes, *r *= 0.11, 0.18, 0.26, 0.25 for measurements 1–4, respectively. The correlation between the four "late" window measurements ranges from 0.54 (ttth1 and ttth3) to 0.91 (ttth3 andttth4). Our goal was to conduct both univariate and multivariate (with related traits) analyses on a chromosome with regions suggestive of linkage, and contrast these methods in the context of genomic screening. Because no candidate chromosomes have been suggested for these jointly modelled phenotypes, we opted to use chromosome 4 as a template due to some replication of linkage findings for EEG [5-7] and its association with the ttth phenotypes.

### FBAT: Background and univariate test statistic

In the first stage of the analysis, we apply our screening technique in a univariate setting to investigate family-based associations with the far frontal left side channel measurements (ttth1). The family-based association test (FBAT) statistic [8] comprises a linear combination of observed offspring genotypes and traits. The test statistic is defined as: *S *= ∑_*ij *_*T*_*ij*_*X*_*ij*_, where *X*_*ij *_denotes a coding of the marker genotype of the *j*^th ^offspring in family *i*. The coded trait of the *j*^th ^offspring in the *i*^th ^family is defined by *T*_*ij*_. In general, the trait will be mean-centered. The test statistic, a score function, in large samples is defined as: *Z *= ∑_*ij *_*T*_*ij*_(*X*_*ij *_- *E*(*X*_*ij*_))/*Var*(*S*)^1/2 ^~ *N*(0,1), where *E*(*X*_*ij*_) is the expected value of the coding function for the offspring genotype, conditional on the parental genotype and parental/offspring phenotypes, which are assumed to follow Mendelian segregation under the null hypothesis. This methodology extends readily to scenarios in which the parental genotypes are not known, using the approach of Rabinowitz and Laird [9]. The FBAT statistic has an additional advantage in that it is robust against population admixture and stratification [9].

### PBAT: Screening methodology

The screening technique proposed by Lange et al. [2] identifies the markers with the highest conditional power [10] without biasing any subsequent test statistic. First, a set of phenotypes (or phenotype) are selected. A model that describes the phenotypes as a function of the genotypes is chosen. When conducting the screening process for the most powerful SNPs for this set of phenotypes, the observed offspring genotypes are replaced by the expected offspring genotypes, conditional on the parental genotypes or sufficient statistics [9]. (The use of the observed genotypes would bias the nominal significance level of the FBAT statistic). This adjusted model is used in estimating effect-size parameters for the genetic model. Lastly, using the conditional power calculations described in Lange and Laird [10], the power for each SNP-trait combination is estimated. The subset of SNPs with the highest conditional power may be selected and their *p*-values need only be adjusted according to the size of the selected subset. This screening process is used in both a univariate and multivariate setting. The process differs only in the model that describes the phenotypes as a function of the genotypes, and thus, how the effect-size estimates are generated (least-squares for univariate, principal components analysis for FBAT-PC [11], and generalized estimating equations for FBAT-GEE [12]. These test statistics and screening methodologies are available in the PBAT software package . The FBAT-PC and FBAT-GEE statistics are briefly reviewed to provide a reference for the reader. Please refer to the original published descriptions [11,12] for further description.

### FBAT: Two approaches to modelling multiple phenotypes

#### FBAT-PC

Using the four-electrode measurements ttth1-ttth4, we construct the "generalized" principal component [11] that maximizes heritability. Heritability is defined as the proportion of the total variance attributable to the genetic effect being tested. This methodology applies generalized principal component analysis to both the phenotypic and genetic variance matrices. This approach differs from standardized generalized principal components analysis, which only applies data reduction techniques to the phenotypic variance matrix. When multiple quantitative phenotypes are measured, a composite phenotype may be constructed which amplifies the phenotype heritability by aggregating the genetic components of all the traits into a single phenotype with maximal heritability. Using the univariate FBAT statistic [8] on this newly created component, FBAT-PC, is particularly attractive, given that higher heritabilities amount to the success of genome-wide association screening.

#### FBAT-GEE

The four electrode measurements, ttth1-tth4, also may be be tested simultaneously using the multivariate FBAT statistic (FBAT-GEE) of Lange et al. [12]. The FBAT-GEE statistic uses generalized estimating equations to analyze multivariate data. The multivariate FBAT-GEE and its validity do not require any assumptions for the phenotypes. It allows for testing of different trait types (e.g., continuous or binary) without specifically modelling the dependence of the multivariate traits on the genotype information. Making effective use of all available trait information, it often leads to increased power to detect the alternative, as it allows for for fewer statistical comparisons by simultaneously testing phenotypes. This is also an attractive methodology, given the increasingly complex traits, such as alcoholism, currently under study.

## Results

### Univariate phenotype: ttth1

Table 1 displays the FBAT test results for ttth1 for five marker-trait combinations with the highest power. The top five power estimates range from 0.9985 to 0.9906. However, the actual power estimates are somewhat arbitrary, because they are dependent upon the predetermined alpha level of the FBAT statistic. Of greater importance is the relative power of the SNP-phenotype association, thus the five markers with the highest overall power were selected. All available Affymetrix SNPs on chromosome 4 were considered in the analysis. Markers with significant Hardy-Weinberg tests (*p*-values < 0.05) were removed, as an indication of genotyping error. Additionally, in genomic association screens, these raw results can no longer be benchmarked against 0.05. For overall significance, using a Bonferroni correction, a *p*-value < 0.01 is required, based upon the reporting of the top five results by power estimate. One of the observed markers, TSC0047225, demonstrates significant association, with a *p*-value of 0.004.

### Multiple phenotypes: ttth1-ttth4

#### FBAT-PC

Of the top five markers with the highest power in the univariate analysis, four (TSC004275, TSC0045785, TSC0570893, and TSC0518297) also have been identified using the principal components statistic. The results are shown in Table 2. Again, only one marker, TSC0047225, achieves significance.

#### FBAT-GEE

Table 3 shows the results from the FBAT-GEE analysis. Interestingly, only two of the five markers (TSC0047225 and TSC0518297) identified in the univariate analysis were among the five demonstrating the most power for this multivariate test. The FBAT-PC and FBAT-GEE results shared only one common SNP (TSC0047225). However, in comparison to both the FBAT and FBAT-PC statistics, the observed significance level for both of these markers increases. TSC0047225 is no longer significant at the 0.01 level, while marker TSC0518297 does not approach the alpha level in either the univariate or multivariate analysis.

## Discussion

Using our screening techniques, we identified a SNP (TSC004275) that is associated with the ttth1-ttth4 phenotypes. This SNP is located at approximately 133 cM on chromosome 4. Concurrent and subsequent analyses will determine whether this finding is supported via other screening methodologies.

Overall, the analysis of these data, using both univariate and multivariate analyses, highlights a key point. More credibility maybe given to markers that are identified through multiple screening techniques. TSC0047225 was selected in all three analyses, and was significant in two of the three (*p *= 0.004). The reason for the similarity in significance levels in the FBAT and FBAT-PC analysis, and the non-significance of the FBAT-GEE result may be due to noise created by a particular phenotypic measurement, or ttth1 maybe primarily responsible for the observed association seen in the univariate analysis. The similarity of the FBAT and FBAT-PC results with regard to the identified SNPs also are surprising, and suggest that the ttth1 phenotype may be driving the association. Those performing future association analyses may want to utilize phenotypes (such as ttth and ECB21 phenotype) that are not as strongly correlated.

Alternatively, there may be an individual locus with pleiotropic effects, and FBAT-PC may simply have more power than FBAT-GEE to detect an association. Using simulated data, Lange et al. [11] demonstrated that FBAT-PC has greater power than FBAT-GEE to detect a causal locus when the phenotypes are highly correlated (*r *= 0.8) and the heritability is constant across phenotypes. In the FBAT-GEE results, the observed heritabilities (not shown) were very similar (~0.4) across phenotypes, and 3 of the 4 ttth phenotypes are highly correlated. Thus, the data suggest a scenario in which the FBAT-PC statistic is likely to have greater power than FBAT-GEE. However, given the similarity of the univariate FBAT and FBAT-PC results, this hypothesis is less likely. Despite the similarity of the FBAT and FBAT-PC results, analysis of multiple phenotypes can be more powerful than that of single phenotypes when attempting to unravel the genetic structure underlying complex traits.

A final possibility is that there is no causal locus, and the finding is spurious. Using any method for genome-wide screening, this is always a possibility, especially when the findings are not supported by other studies. However, the paper by Van Steen et al. [1] demonstrated via simulation studies that the nominal alpha levels are maintained using these screening tools. Association testing for genome-wide screening is novel, and more time is needed to determine whether initial results will be replicated using this testing methodology.

## Conclusion

In this paper, we implemented a novel screening technique in the context of genome-wide association testing, and contrasted the results of this screening methodology across univariate and multivariate settings. Through this analysis, it was suggested both univariate and multivariate testing methodologies are useful in detecting genotype × phenotype associations. Additionally, multivariate methods can be powerful tools in finding associations that may not be detectable in the univariate setting.

## Abbreviations

COGA: Collaborative Study on the Genetics of Alcoholism

EEG: Electroencephalogram

FBAT: Family-based association testing

GEE: Generalized estimating equation

PBAT: Pedigree-based association testing

PC: Principle components

SNP: Single-nucleotide polymorphism

## Authors' contributions

AM conducted the association analysis and drafted the manuscript. MBM assisted in drafting the manuscript. JS participated in the study design and analysis. PK participated in the design and coordination of the study. RL assisted with study design. NML helped conceive the study and participated in its coordination. CL helped conceive the study and conducted portions of the association analysis. KVS conceived the study, provided additional association analyses, and aided in drafting the manuscript.

## Figures and Tables

**Table 1:** FBAT results using ttth1

Marker	Allele frequency	Allele	No. Informative families	*p*-Value FBAT	Power^a^
TSC1091705	1	0.5581	44	0.2593	0.9985
TSC0047225	2	0.6268	47	0.0040	0.9964
TSC0149925	1	0.4551	42	0.0575	0.9948
TSC0274878	2	0.4111	42	0.0256	0.9908
TSC0518297	1	0.4477	38	0.8866	0.9906

Analysis restricted to additive genetic model.

^a^Conditional power calculation for the FBAT test with alpha level 10^-8^.

**Table 2:** FBAT-PC results using ttth1-ttth4

Marker	Allele	Allele frequency	No. Informative families	FBAT-PC	Power^a^
TSC1091705	1	0.5581	44	0.2761	0.9991
TSC0274878	2	0.4111	42	0.0286	0.9984
TSC0047225	2	0.6268	47	0.0040	0.9975
TSC0149925	1	0.4551	42	0.1015	0.9965
TSC0059981	1	0.5495	38	0.4511	0.9914

Analysis restricted to additive genetic model.

^a^Conditional power calculation for the FBAT test with alpha level 10^-8^.

**Table 3:** FBAT-GEE results using ttth1-ttth4

Marker	Allele	Allele frequency	No. Informative families	*p*-Value FBAT-GEE	Power^a^
TSC0047225	2	0.6268	47	0.0526	0.9652
TSC0045785	2	0.6545	35	0.7628	0.9067
TSC0570893	1	0.3989	46	0.4014	0.9039
TSC0518297	1	0.4477	38	0.5206	0.9011
TSC1082961	1	0.4500	37	0.2102	0.8685

Analysis restricted to additive genetic model.

^a^Conditional power calculation for the FBAT test with alpha level 10^-8^.
